# Drip, Ship, and On-Demand Endovascular Therapy for Acute Ischemic Stroke

**DOI:** 10.1371/journal.pone.0150668

**Published:** 2016-03-03

**Authors:** Man-Seok Park, Woong Yoon, Joon-Tae Kim, Kang-Ho Choi, Seung-Ho Kang, B. Chae Kim, Seung-Han Lee, Seong-Min Choi, Myeong-Kyu Kim, Ji-Sung Lee, Eun-Bin Lee, Ki-Hyun Cho

**Affiliations:** 1 Department of Neurology, Chonnam National University Medical School, Gwangju, Korea; 2 Department of Radiology, Chonnam National University Medical School, Gwangju, Korea; 3 Department of Neurology, Chonnam National University Hwasun Hospital, Hwasun, Korea; 4 Gwangju-Jeonnam Regional Cerebrovascular Center, Chonnam National University Hospital, Gwangju, Korea; 5 Clinical Research Center, Asan Medical Center, Seoul, Korea; University of Glasgow, UNITED KINGDOM

## Abstract

**Background:**

The “drip and ship” approach can facilitate an early initiation of intravenous thrombolysis (IVT) for acute ischemic stroke (AIS) at community hospitals. New endovascular treatment modalities, such as stent retrieval, have further improved the rate of safe and successful recanalization. We assessed the clinical outcomes of on-demand endovascular therapy in patients with AIS who were transported to a comprehensive stroke center under the “drip and ship” paradigm.

**Methods:**

This retrospective study evaluated prospectively registered patients with acute large vessel occlusions in the anterior circulation who underwent endovascular recanalization after IVT at our regional comprehensive stroke center between January 2011 and April 2014. Clinical outcomes and neuroradiological findings were compared between patients who received IVT at the center (direct visit, DV) and at a community hospital (drip and ship, DS).

**Results:**

Baseline characteristics such as age, initial National Institutes of Health Stroke Scale (NIHSS) score, and risk factors for stroke were similar, and most patients underwent endovascular therapy with a Solitaire stent (81.9% vs. 89.3% for DV and DS, respectively, *P* = 0.55). The average initial NIHSS score was 12.15±4.1 (12.06 vs. 12.39 for DV and DS, respectively, *P* = 0.719). The proportions of long-term favorable outcomes (modified Rankin Scale score ≤2 at 90 days) and successful recanalization (Thrombolysis in Cerebral Ischemia score ≥2b) were not significantly different (*P* = 0.828 and 0.158, respectively). The mortality rates and occurrences of symptomatic intracerebral hemorrhage were not significantly different (*P* = 0.999 and 0.267, respectively).

**Conclusions:**

The “drip and ship” approach with subsequent endovascular therapy is a feasible treatment concept for patients with acute large vessel occlusion in the anterior circulation that could help improve clinical outcomes in patients with AIS.

## Introduction

Untreated acute occlusion of a large cerebral artery has a poor prognosis. However, early recanalization using intravenous thrombolysis (IVT), intra-arterial (IA) endovascular treatment, or a combination of these therapies can reduce occlusion-associated mortality and lead to improved patient outcomes. IVT can be easily performed at community hospitals, facilitating an advantageous early initiation of thrombolytic recanalization therapy. IA endovascular therapy has a higher probability of successful recanalization than IVT, but it is not widely available outside of specialized stroke centers[1–3].The combined use of IVT and IA endovascular therapy to treat acute occlusion appears preferable because it allows an early initiation of thrombolytic therapy and results in a high recanalization rate. Previous studies on endovascular treatment modalities (IMS-III, MR RESCUE, and SYNTHESIS trials) did not show that endovascular treatment had any additional clinical benefit in comparison with standard IVT[4–6]. However, each of these three studies had critical limitations such as routine use of wrong instruments and techniques and imaging protocols that were unsuitable for proper patient selection. Recent multicenter, open-label, randomized controlled trials (MR CLEAN, ESCAPE, SWIFT PRIME, EXTEND-IA, and REVASCAT) support the superiority of endovascular treatment incorporating the use of second-generation mechanical thrombectomy devices (stent retrievers) as the best medical treatment with or without IV-tPA for an acute occlusion in the anterior circulation of a large cerebral artery[7–11]. New evidence from these studies has proven that substantial benefit is derived from the use of newer endovascular treatment methods and supports new standards for acute ischemic stroke (AIS) management.

The “drip and ship” (DS) paradigm refers to the use of IV-tPA as the initial treatment of choice in smaller community and rural hospitals with subsequent patient transportation to regional comprehensive stroke centers (CSCs). Several reports have demonstrated the feasibility and safety of this AIS treatment approach and that it also increases regional tPA treatment rates among patients with AIS[12–15]. According to the DS paradigm, patients with an acute ischemic stroke begin receiving intravenous tPA at a community hospital and are then transferred to a regional CSC for further management of their condition, including post-thrombolysis neurointensive monitoring, endovascular treatment, and neurosurgical procedures.

In 2008, the Ministry of Health and Welfare (MHW) of South Korea initiated a regional CSC program to decrease stroke incidence and mortality. CSCs were established in nine provincial regions nationwide[16]. In 2010, our hospital was designated as a regional CSC, serving approximately 4.5 million people residing in this province. According to the DS approach, community hospitals in our region initiate IVT for patients with AIS and then quickly transport them to our CSC. On-demand endovascular recanalization treatment is considered for patients transported from community hospitals if they exhibit persistent neurologic deficits despite IVT. In this study, we analyzed the prospective stroke registry at our hospital to assess clinical outcome patterns following endovascular recanalization treatment in patients with AIS who were transported to our hospital under the DS paradigm. Because the Solitaire device has been the first-line treatment choice for endovascular recanalization at our hospital since 2011, we also evaluated the clinical outcomes of stent retrieval using this device in this patient cohort.

## Methods

### Patients

As a regional CSC, our hospital works closely with its neighboring community hospitals. Whenever endovascular therapy is considered, the community hospitals promptly transfer patients to our treatment center. We retrospectively reviewed the records of patients with AIS who underwent the combination treatment comprising IV-tPA and endovascular therapy between January 2011 and April 2014. The analysis included only patients with an acute large arterial occlusion in the anterior circulation, with the proximal middle cerebral artery (pMCA) and internal carotid artery (ICA) as the main occlusion sites. Two different patient groups were derived from the acute ischemic stroke database at our hospital based on where the IV-tPA was initiated. Patients in the “direct visit” (DV) group fulfilled the following inclusion criteria: (1) primary admission at our stroke center within 4.5 h after the estimated time of pMCA or ICA occlusion, (2) acute occlusion confirmed by magnetic resonance angiography (MRA) or conventional angiography, and (3) subsequent endovascular therapy initiated if there was no recovery from neurological deficits present after urgent IV-tPA at our stroke center. Patients in the DS group fulfilled the following inclusion criteria: (1) primary admission at a community hospital, (2) IV-tPA initiated at the community hospital, (3) referral and admission to our stroke center within 6 h after the estimated time of pMCA or ICA occlusion, (4) acute occlusion confirmed by MRA or conventional angiography, and (5) on-demand endovascular therapy owing to the lack of recovery.

On admission, a stroke neurologist conducted a neurological assessment based on the National Institutes of Health Stroke Scale (NIHSS). All patients underwent a non-enhanced brain computed tomography (CT) scan and multimodal magnetic resonance imaging (MRI) prior to endovascular treatment. In the DS group, all patients underwent brain CT at the community hospital and subsequent brain MRI was performed at our stroke center following transfer. If MRI was performed at the community hospital within 1 h prior to arrival at our stroke center, the results of that MRI were used in the selection of patients for endovascular therapy. The inclusion criteria for endovascular therapy were as follows: (1) presentation within 6 h of stroke onset, (2) no evidence of intracranial hemorrhage on brain CT or MRI, (3) major arterial occlusion on MRA or conventional angiography, (4) a target mismatch pattern on multimodal MRI according to visual estimation [time-to-peak map of perfusion imaging showing a lesion volume ≥30% larger than that detected using diffusion-weighted imaging(DWI)], (5) infarct volume on DWI or non enhanced CT less than one-third of the MCA territory, and (6) premorbid modified Rankin Scale (mRS) score ≤ 3.

### Ethics Statement

This study was approved by the Institutional Review Board (IRB) of Chonnam National University Hospital. All of the clinical investigations described in this study were conducted in accordance with the principles expressed in the Declaration of Helsinki. Written informed consent was obtained from each patient or a family member.

### Endovascular recanalization treatment

All endovascular therapies were performed by an experienced interventional neuroradiologist. Cerebral angiography and endovascular therapy was performed using a femoral approach under local anesthesia. In all patients, first line treatment used a Solitaire stent (20-mm long and 4-mm wide; eV3 Inc., Irvine, CA) for endovascular therapy. When endovascular mechanical recanalization using a Solitaire stent was unsuccessful, additional endovascular procedures were performed, including aggressive clot disruption with a microwire, balloon angioplasty, or aspiration thrombectomy. Stent-retriever thrombectomy was not performed using balloon guide or distal access catheters. The time of endovascular recanalization treatment initiation was defined as the time of initial arterial puncture. Recanalization status was assessed based on the final angiogram and classified according to the Thrombolysis in Cerebral Ischemia (TICI) scale[17]. A successful recanalization was defined as a TICI grade of 2b or 3. Angiographic collateral grade was evaluated using the American Society of Interventional and Therapeutic Neuroradiology/Society of Interventional Radiology (ASITN/SIR) Collateral Flow Grading System based on baseline angiography[18]. This angiographic scale assigns patients to grade 0, no collaterals visible at the ischemic site; 1, slow collaterals to the periphery of the ischemic site with partial defect persistence; 2, rapid collaterals to the periphery of the ischemic site with persistence of some of the defect and to only a portion of the ischemic territory; 3, collaterals with slow but complete angiographic blood flow to the ischemic bed by the late venous phase; and 4, complete and rapid collateral blood flow to the vascular bed in the entire ischemic territory by retrograde perfusion. Dichotomization of poor and good collaterals was accomplished by dividing patients into groups of grades 0–2 vs. grades 3–4. Consensus assessment of the angiographic findings was performed by two stroke neurologists who were blinded to the procedure.

### Long-term outcomes and safety

Patient NIHSS scores were assessed at baseline and at hospital discharge and differences in group mean scores at each time point were statistically compared. Patient mRS at 90 days after stroke onset was also obtained and compared between the two treatment groups. A long-term favorable outcome was defined as mRS of 0–2 at 90days after treatment. Safety assessments included monitoring for the potential development of symptomatic intracranial hemorrhage (sICH) or hemorrhagic transformation (HT). HT was categorized as a hemorrhagic infarction (type 1 or 2) or parenchymal hematoma (PH) (type 3 or 4) according to the descriptions from the European Cooperative Acute Stroke Study (ECASS) I and II[19, 20]. sICH was defined as any increase from baseline in NIHSS score. HT or sICH was assessed by means of a follow-up brain CT or MR gradient echo imaging.

### Statistical analysis

Continuous variables are reported as mean (±standard deviation, SD) and categorical values are reported as the number (%) of patients. Patient baseline DV and DS group characteristics and clinical outcomes were compared using Pearson’s chi-squared test, Fisher’s exact test, or Student *t*-tests, as appropriate, for each variable. The results of comparisons are presented either as the mean or the percent difference between two groups with 95% CIs.

The Cochran–Mantel–Haenszel (CMH) shift test and ordinal logistic regression analysis were used to evaluate mRS at 90 days within the DS group, with the effects of the DS group reported as common ORs with 95% CIs for a shift in the direction of the lower mRS score at 90 days (indicating a favorable outcome). In multivariate ordinal logistic regression, predetermined variables were chosen as potential confounders for statistical adjustment. These selected variables were age, stroke severity (NIHSS score) at baseline, time from stroke onset to groin puncture, atrial fibrillation, diabetes mellitus, and the occlusion of the internal carotid artery terminus.

A 2-sided *P* value of <0.05 was considered statistically significant. All statistical analyses were performed using SPSS version 21 (IBM Corp., Armonk, NY, USA) and SAS version 9.4 (SAS Institute, Cary, NC, USA).

## Results

### Patient characteristics

During the study period, 105 patients with radiologically confirmed pMCA or ICA occlusion were treated with IV-tPA followed by endovascular therapy at our stroke center. Within this group, 77 patients received IVT at our stroke center’s emergency department (DV group). For the remaining 28 patients, IV-tPA was initiated at a community hospital prior to patient transfer to our stroke center (DS group). Within the DS group, 23 patients underwent brain MRI at our stroke center. Most patients were transported by ambulance with no associated complications. Patient characteristics such as age, gender, stroke risk factors, and stroke subtypes were comparable between the treatment groups. NIHSS scores prior to endovascular therapy were not significantly different between the two groups (12.06 vs. 12.39 for DV and DS, respectively, *P* = 0.719) (Table 1). All patients received a full dose of IV-tPA (0.9mg/kg), and the Solitaire stent was the primary option used for endovascular therapy (81.9% vs. 89.3% for DV and DS, respectively, *P* = 0.55). Other endovascular treatment modalities such as ICA or MCA stent, balloon angioplasty, and aspiration thrombectomy were also used at similar frequencies in each group. The Solitaire stent was not used if successful recanalization was achieved with acute carotid artery stenting for proximal ICA occlusion (DV, 13%; DS, 3.6%), balloon angioplasty (DV, 5.2%; DS, 3.6%), or aspiration thrombectomy (DV, 1.3%; DS, 3.6%). The mean±SD time from stroke onset to arterial puncture in the DS group was significantly longer than that in the DV group (300±63.3 min vs. 219.2±55.9 min, *P* = 0.001)(Table 1). The time from stroke onset to successful recanalization was also significantly delayed in the DS group (*P* = 0.001).

**Table 1:** Patient Characteristics at Baseline.

	Direct Visit (n = 77)	Drip and Ship (n = 28)	Mean or Percentage Difference (95% CI)	P-value†
**Age, mean ± SD (y)**	69.6 ± 10.3	67.9 ± 10.1	1.7 (-2.8, 6.2)	0.451
**Male gender, n (%)**	49 (63.6)	16 (57.1)	6.5 (-15.2, 27.9)	0.545
**Risk factors, n (%)**				
** Hypertension**	45 (58.4)	16 (57.1)	1.3 (-20.3, 22.9)	0.905
** Hyperlipidemia**	12 (15.6)	8 (28.6)	-13.0 (-34.1, 8.8)	0.134
** Diabetes mellitus**	13 (16.9)	6 (21.4)	-4.5 (-26.0, 17.2)	0.593
** Smoking**	19 (24.7)	8 (28.6)	-3.9 (-25.4, 17.8)	0.686
** Atrial Fibrillation**	35 (45.5)	12 (42.8)	2.6 (-19.1, 24.1)	0.813
**Stroke etiology, n (%)**				0.539
** LAD**	19 (24.7)	5 (17.9)	6.8 (-14.9, 28.2)	
** CE**	36 (46.8)	12 (42.9)	3.9 (-17.8, 25.4)	
** UD**	22 (28.6)	11 (39.3)	-10.7 (-31.9, 11.0)	
**Initial NIHSS score**	12.06 ± 4.05	12.39 ± 4.31	-0.33 (-2.13, 1.48)	0.719
**Time to stroke center, min**	95.0 ± 49.8	204.86 ± 56.7	-109.8 (-132.4, -87.2)	< .001
**Onset to puncture, min**	219.2 ± 55.9	300 ± 63.3	-80.8 (-106.2, -55.5)	< .001
**Target artery, n (%)**				
** ICA**	32 (41.6)	11 (39.3)	2.3 (-19.4, 23.8)	0.834
** proximal**	23 (29.9)	6 (21.4)	8.4 (-13.3, 29.7)	0.392
** distal**	11 (14.3)	6 (21.4)	-7.1 (-28.5, 14.6)	0.382
** MCA**	53 (68.8)	21 (75)	-6.2 (-27.6, 15.6)	0.54
** Tandem occlusion**	10 (13.0)	5 (17.9)	-4.9 (-26.3, 16.9)	0.538
**Mechanical thrombectomy, n (%)**				
** Solitaire**	63 (81.9)	25 (89.3)	-7.5 (-28.8, 14.3)	0.55
** Intracranial stent**	2 (0.26)	1 (3.6)	-1.0 (-22.5, 20.7)	>.999
** Balloon angioplasty**	7 (9.1)	6 (21.4)	-12.3 (-33.4, 9.5)	0.103
** Aspiration thrombectomy**	14 (18.2)	9 (32.1)	-14.0 (-35.0, 7.9)	0.126
** Carotid stent**	19 (24.3)	4 (14.3)	10.4 (-11.4, 31.6)	0.255

† P-values are calculated by Pearson chi-square test, Fisher's exact test or Student's t-test as appropriate

LAD indicates large artery disease; CE, cardioembolism; UD, undetermined; NIHSS, NIH Stroke Scale; ICA, internal carotid artery; MCA, middle cerebral artery; ED, emergency department.

### Functional outcomes and neuroradiological findings

Of the 105 patients, 74 (70.5%) had occlusion of the MCA and 44 (40.1%) had occlusion of the ICA. Both groups had similar proportions of occluded target arteries (ICA, 41.6% vs. 39.3% in the DV and DS groups, respectively, *P* = 0.999; MCA, 68.8% vs. 75% in the DV and DS groups, respectively, *P* = 0.633) (Table 1). In all patients, the rate of successful recanalization, defined as TICI ≥2b, was 89.6%, with a comparable proportion of successful recanalization rates in each group (92.2% vs. 82.1% in the DV and DS groups, respectively, *P* = 0.158) (Table 2). The proportion of patients with poor pretreatment collaterals (48.1% vs. 53.6%, respectively, *P* = 0.663) and NIHSS scores at baseline (12.1 vs. 12.4, respectively, *P* = 0.719) were also similar between DV and DS.

**Table 2:** Clinical Outcomes and Neuroradiological Findings.

	Direct Visit (n = 77)	Drip and Ship (n = 28)	Mean or Percentage Difference (95% CI)	P-value†
**Total recanalization (TICI 2b or 3), n (%)**	71 (92.2)	23 (82.1)	10.1 (-11.7, 31.3)	0.158
**NIHSS score (discharge), mean ± SD**	7.22 ± 5.96	6.71 ± 4.71	0.51 (-1.97, 2.98)	0.686
**mRS score (90 days), mean ± SD**	2.79 ± 1.82	2.86 ± 1.82	-0.06 (-0.86, 0.73)	0.872
**Favorable long-term outcome, n (%)**				
** 90 days mRS 0–1**	18 (23.4)	5 (17.9)	5.5 (-16.1, 27.0)	0.545
** 90 days mRS 0–2**	39 (50.6)	13 (46.4)	4.2 (-17.4, 25.7)	0.702
**Mortality (90 days), n (%)**	8 (10.4)	2 (7.1)	3.2 (-18.4, 24.7)	>.999
**sICH, n (%)**				
** sICH (ΔNIHSS ≥ 1)**	12 (15.6)	7 (25.0)	-9.4 (-30.7, 12.3)	0.268
** sICH (ΔNIHSS ≥ 4)**	4 (5.2)	3 (10.7)	-5.5 (-27.0, 16.1)	0.38
**Hemorrhagic transformation, n (%)**				
** HI-1**	15 (19.5)	6 (21.4)	-1.9 (-23.5, 19.7)	0.825
** HI-2**	11 (14.3)	8 (28.6)	-14.3 (-35.4, 7.5)	0.093
** PH-1**	11 (14.3)	2 (7.1)	7.1 (-14.6, 28.5)	0.506
** PH-2**	2 (2.6)	3 (10.7)	-8.1 (-29.5, 13.6)	0.117

† P-values are calculated by Pearson chi-square test, Fisher's exact test or Student's t-test as appropriate

TICI indicates thrombolysis in cerebral ischemia; mRS, modified Rankin scale; sICH, symptomatic intracerebral hemorrhage; HI, hemorrhagic infarction; PH, parenchymal hematoma; ED, emergency department

**Fig 1:**
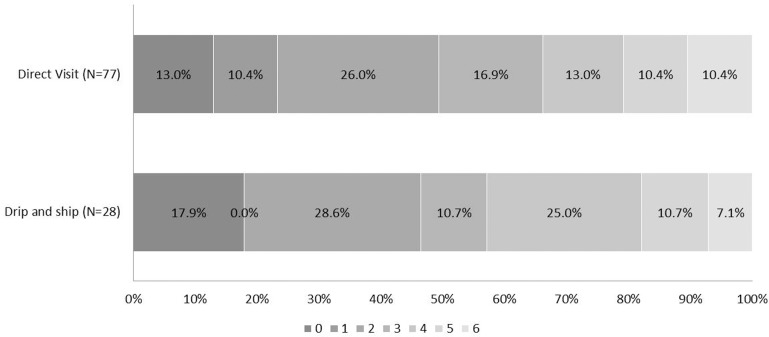
Distribution of mRS score at 90 days after IV-tPA and IA endovascular treatment. Individuals with a mRS score of 0, 1, or 2 are considered to be functionally independent. The proportion of favorable outcomes at 90 days was comparable between the groups (50.6% vs. 46.4% for DV and DS, respectively, *P* = 0.702). There was no significant difference between the two groups in the overall distribution of mRS score in a shift analysis with univariate ordinal regression (OR 0.90, 95% CIs 0.42–1.92, *P* = 0.782) or after adjustment for the treatment effects of age, stroke severity (NIHSS score) at baseline, time from stroke onset to arterial puncture, atrial fibrillation, diabetes mellitus, and the occlusion of the internal carotid artery terminus multivariate ordinary logistic regression (OR 0.82, 95% CIs 0.27–2.46, *P* = 0.719).

NIHSS scores at discharge were not different between the DV and DS groups (7.22 vs. 6.71, respectively, *P* = 0.686). The average mRS at 90 days (2.79 vs. 2.86 for DV and DS, respectively, *P* = 0.872) and the proportion of favorable outcomes at 90 days (mRS ≤2) (50.6% vs.46.4% for DV and DS, respectively, *P* = 0.702) were also comparable (Table 2). There was no significant difference between groups in the distribution of mRS scores at 90 days with unadjusted shift analysis (OR 0.90, 95% CIs 0.42–1.92, *P* = 0.782) or adjusted shift analysis (OR 0.82, 95% CIs 0.27–2.46, *P* = 0.719) (Fig 1).

Concerning safety, the frequency of HT development (50.6% vs. 67.9% for DV and DS, respectively, *P* = 0.128) and HT of parenchymal hematoma (PH) type occurrence rates (PH-1, 14.3% vs. 7.1% for DV and DS, respectively, *P* = 0.506; PH-2, 2.6% vs. 10.7% for DV and DS, respectively, *P* = 0.117) were not significantly different between groups. Mortality rates (10.4% vs. 7.1% for DV and DS, respectively, *P* = 0.999) and sICH occurrence (15.6% vs. 25% for DV and DS, respectively, *P* = 0.267) were not significantly different between groups (Table 2).

**Fig 2:**
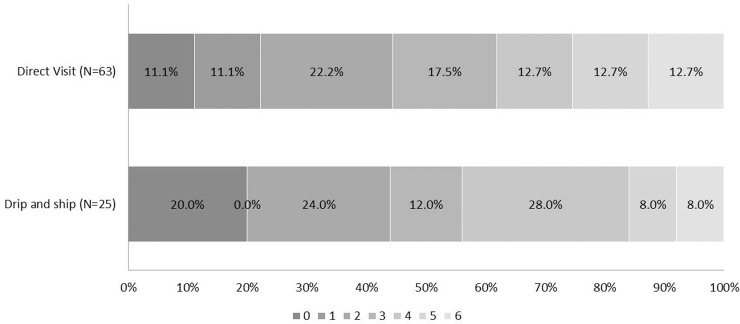
Distribution of mRS score at 90 days after IV-tPA and Solitaire stent retrieval. Persons with a mRS score of 0, 1, or 2 are considered to be functionally independent. The proportion of favorable outcomes at 90 days was comparable between the two groups (44.4% vs. 44.0% for DV and DS, respectively, *P* = 0.581). There was no significant group difference in the overall distribution of mRS scores in a shift analysis with univariate ordinal regression (OR 1.09, 95% CIs 0.48–2.46, *P* = 0.833) as well as after adjustment of the treatment effects of age, stroke severity (NIHSS score) at baseline, time from stroke onset to arterial puncture, atrial fibrillation, diabetes mellitus, and the occlusion of the internal carotid artery terminus in an analysis of multivariate ordinal logistic regression (OR 0.99, 95% CIs 0.31–3.14, *P* = 0.983).

When analyzing the cohort of patients treated with Solitaire stent retrieval, mean mRS scores at 90 days were comparable between the groups (44.4% vs.44.0% for DV and DS, respectively, *P* = 0.581). The distribution of mRS scores at 90 days was not significantly different between the two groups with unadjusted shift analysis (OR 1.09, 95% CIs 0.48–2.46, *P* = 0.833) or adjusted shift analysis (OR 0.99, 95% CIs 0.31–3.14, *P* = 0.983) (Table 2, Fig 2). Successful recanalization rates, mortality rates, and sICH and HT proportions were also similar in both Solitaire stent retrieval groups (data not shown).

## Discussion

When treating acute large arterial occlusion in the anterior circulation, early recanalization is one of the most important factors for achieving a favorable clinical outcome. The DS paradigm is expected to increase the number of patients who may benefit from IV-tPA treatment. Tekle et al. reported that the DS paradigm was used for 17% of all patients treated with IV-tPA for AIS in the United States, with those patients achieving significantly higher rates of home discharge/self-care in comparison with patients treated with IV-tPA at the time of primary emergency department arrival[14]. However, IV-tPA has only a modest treatment effect on AIS resulting from large arterial occlusion because it has a low potential for recanalizing vessels that are occluded by a large thrombus. Therefore, after rapid transfer to a CSC, rescue endovascular therapy should be considered for patients who have a large vessel visibly occluded by a big clot on the initial MRI/CT performed at a community hospital. In the present study, the rates of long-term favorable outcome and sICH were similar between the DS and DV groups. No complications occurred during transportation, and the development of sICH was rare overall. These results were also comparable to the outcomes reported from recent successful trials of endovascular therapy[2,3,11]. Endovascular therapy is not available at most community hospitals. By improving the network between community hospitals and regional CSCs, more patients with AIS could benefit from early IV-tPA initiation and the opportunity for rescue endovascular therapy. If community hospitals use the same imaging protocol for CT, CTA, and MRI and these images are transferred to comprehensive stroke centers before referring stroke patients, these centers would be able to perform the optimal and individualized management for every single stroke patient from the very beginning of the diagnostic and therapeutic pathway.

The high recanalization success rate in our study can be attributed to various factors such as the prevalent use of a new endovascular device and trained interventional team. The Solitaire stent was used in 83.8% of the patients included in this study. This new device has been reported to increase successful recanalization rates and reduce total IA treatment time. For this reason, the Solitaire stent has been increasingly used to treat AIS caused by intracranial large vessel occlusion. We previously reported favorable clinical and angiographic outcomes after mechanical thrombectomy with the Solitaire stent in patients with distal intracranial ICA occlusion[21]. One of the most significant benefits of this stent-retriever is that blood flow starts to be restored as soon as the device makes contact with the blood clot.

sICH is the major adverse event in IV thrombolysis or IA treatment after standard-dose (0.9 mg/kg) IV-tPA. For this reason, low dose tPA (0.6 mg/kg) was used to reduce the risk of sICH in previous IV/IA combined treatment studies[22–24]. However, we previously found that a standard dose of tPA administered at community hospitals with subsequent IA treatment using urokinase at the CSC did not increase the risk of ICH[25]. Shaltoni et al. [26] also demonstrated that IA treatment after standard-dose IV-tPA given to patients with persistent occlusion and/or lack of clinical improvement appears as safe as IV-tPA alone or low-dose IV-tPA followed by IA treatment. In the current study, all patients were administered standard-dose IV-tPA as an initial treatment and both groups produced a low incidence of sICH and mortality, which was in accordance with our previous work. These results suggest that standard-dose IV-tPA (0.9 mg/kg) may be used safely at community hospitals before subsequent endovascular therapy, which would be particularly useful in supporting the DS approach.

This study evaluated patients with moderately severe disease. The mean±SD initial NIHSS score for patients in this study was 12.2±4.1, which was lower than that in recent endovascular trials. Considering the moderate severity of stroke, the proportion of good clinical outcomes was relatively low in this study. Therefore, we analyzed baseline characteristics that may negatively impact functional outcomes such as the proportion of elderly patients (>80 years) and those with a DWI-Alberta Stroke Program Early CT score (ASPECTS) of <5. The mean±SD age of patients in this study was 69.2±10.2 years, which was not different from the age reported in recent successful endovascular trials (SWIFT ≒ 67, ESCAPE ≒ 70, and MR CLEAN ≒ 65). Gratz et al. revealed that patients <80-year old had high rates of achieving a favorable outcome and few periprocedural complications[27]. There were 11 (10.5%) patients ≥80-year old in this study, which may have negatively impacted clinical outcomes. However, the proportion of octogenarians was similar in the current study and the recent successful endovascular trials. Patients with extensive ischemic lesions are known to respond poorly to revascularization treatment. Because we selected patients using either perfusion–diffusion mismatch or diffusion-clinical mismatch, patients with low DW-ASPECTS could be included in this study. We assessed DW-ASPECTS retrospectively in the 98 patients for whom DWI was performed prior to endovascular treatment. There were 19 (19.4%) patients in this study with extensive pretreatment ischemic lesions as indicated by a DWI-ASPECTS of <5. This frequency may have negatively impacted the functional outcomes. When we compared the patients with low and high DWI-ASPECTS, the HT rate was significantly higher in those with low DWI-ASPECTS. However, for mRS at 90 days, the mortality and sICH rates were not different between the low and high DWI-ASPECTS groups. Balloon guide catheters and distal access catheters were not used in this study during stent-retriever thrombectomy. This condition may have also contributed to the overall relatively low frequency of good clinical outcome.

Although the present results were similar between treatment groups, one major question remained unanswered. The time from stroke onset to arterial puncture was significantly longer in the DS group than in the DV group. We already know that the time from stroke onset to recanalization is critical to the success of endovascular therapy. Nevertheless, study results showed similar clinical outcome profiles in both groups, possibly owing to the careful selection of patients for on-demand endovascular therapy. During the study period, 185 patients were referred from community hospitals after IV-tPA and 48 (25.9%) were treated with endovascular mechanical thrombectomy. There were 28 anterior large artery occlusion cases and 20 posterior circulation artery occlusion cases. The remaining 137 (74.1%) patients were excluded from subsequent endovascular therapy owing to the lack of a target artery occlusion, non-accessible distal artery occlusion, large infarct core on DWI, or rapid recovery with recanalization of the occluded vessel after IV-tPA. Of the 137 patients, 48 (35%) had no perfusion–diffusion mismatch with a large infarct core on the qualifying image in our stroke center. This subgroup may help explain why there was no difference in group clinical outcomes despite the delay of more than 1 h in the DS group in comparison with the DV group. The mean±SD baseline NIHSS score for the DS group was 12.39±4.31. Moderate stroke severity despite large vessel occlusion during the “drip and ship” approach may indicate that only patients with good collaterals and small DWI lesions have been treated. This limit may argue for there being a selection bias in the DS group. We may have selected those patients who could be stabilized with penumbra and clinical deficits over 90 min or who had good collaterals. However, there was no significant difference in the proportion of patients with poor collaterals between treatment groups. In the SWIFT trial, 67% of patients had poor pretreatment collaterals, which did not exceed the proportion of patients with poor collaterals in the present study. There is no entirely satisfactory explanation for as to why clinical outcomes were not different between treatment groups. Furthermore, the study population was too small to fully address this main question. This may explain why there was no difference in clinical outcome, HT rate, and sICH rate between the two groups despite the >90 min difference in the time of endovascular treatment initiation. In addition, there were several other limitations to this study, including its retrospective study design and the exclusion of posterior circulation artery and MCA branch occlusion. Finally, endovascular recanalization treatment was conducted by an expert stroke team; thus, the present results may not be generalizable to other clinical settings.

In conclusion, “Drip, ship, and on-demand endovascular therapy” may be a feasible treatment paradigm for patients with acute large arterial occlusion in the anterior circulation. Careful patient selection with advanced neurovascular imaging and the use of new endovascular devices such as a stent-retriever device may help minimize the occurrence of adverse events during recanalization treatment. However, large-scale randomized prospective trials are required to confirm the expected impact of “drip, ship, and on-demand endovascular therapy” on clinical outcomes.
